# Effect of Force Level and Gender on Pinch Force Perception in Healthy Adults

**DOI:** 10.1177/2041669520927043

**Published:** 2020-05-20

**Authors:** Lin Li, Yanxia Li, Huihui Wang, Wenqi Chen, Xinyu Liu

**Affiliations:** Department of Physical Education, Renmin University of China; College of Physical Education, Langfang Teachers University, Hebei, China; School of Physical Education, Zunyi Medical University, Guizhou, China; School of Sociology and Population Studies, Renmin University of China

**Keywords:** pinch, force perception, force reproduction task, proprioception

## Abstract

This study investigated the effects of both force level and gender on pinch force reproduction errors in normal participants during an ipsilateral force reproduction task. In total, 20 healthy participants were asked to generate a range of levels of reference forces ranging from 5% to 60% maximal voluntary isometric contraction (MVIC) in increments of 5% MVIC using the tip pinch and to reproduce these forces using the same hand. The results showed that the males more accurately and consistently reproduced the forces than did the females, and the most accurate estimation occurred at approximately 20% to 35% MVIC. This finding can help us better understand the reasons for the higher rate of musculoskeletal disorders in females than in males and to develop tools and preventive strategies to decrease the rate of hand injuries in both genders.

## Introduction

Pinching tasks with different levels of force are frequently used in workplaces. Workers with various occupations, such as mechanics, repair persons, and engineers, must maintain different constant, submaximum pinch grip forces using different hand tools when performing a wide range of operations, from assembling small electronics or larger aircraft. Appropriately gripping an object requires complex motor coordination in order to ensure that an appropriate level of pinch force be applied such that an object does not slip and is not crushed. Excess force application can also cause user fatigue, and repeated or unnecessary pinch forces have been previously identified as being linked to musculoskeletal disorder (MSD) development (Birkbeck & Beer, 1975; Feldman et al., 1983; Silverstein et al., 1986, 1987). The pinch forces repeatedly generated in the workplace may be slightly higher than necessary, which is especially important for MSD, as injuries are primarily the result of minor traumas incurred over extended time periods. Repeated and unnecessarily high pinch forces might induce minimal but long-term stress in some structures and thus a significant injury (Vafadar et al., 2015).

A variety of hand tools such as clamps, wrenches, or pliers are commonly employed in a range of professions to grip and apply force to particular objects. The level of force exerted with the pinch grip was a key consideration during the design and use of such tools. The level of force exerted has an impact on the force perception of a given individual. To determine the optimal level of force for accurately sensing the pinch force and to design tools and preventive approaches that reduce the risk of injury due to repeated and unnecessarily high pinch forces, it is critical that the perception of forces of different levels is understood. Previous studies (Jackson & Dishman, 2000; Jones & Hunter, 1982; Kumar & Simmonds, 1994; Walsh et al., 2011; West et al., 2005) have demonstrated that moderate forces (40% or 50% maximal voluntary isometric contraction [MVIC]) can be estimated with the highest accuracy. In contrast to other joints in the human body, the fingers are controlled with greater precision by the tendons attached to the muscles. Because finger movements require the coordination of many tendons, which are generally not as oxygenated as muscles, they easily lead to fatigue. Therefore, fingers cannot typically maintain a high target force. Appropriate sensation and force control is dependent upon accurately coordinating the two fingers (thumb and index finger) involved in the pinch grip. As force increases, the difficulty of pinch grip coordination increases. Thus, our hypothesis was that the most accurate estimation occurs for lower force levels in pinch grip compared with other joints.

It was necessary to investigate pinch force perception in females as a risk factor associated with MSD development. Prior research has shown that MSD development is subject to gender differences, with females having a higher risk than males with the same occupation (Bovee et al., 1993; McCurdy et al., 1989; Treaster & Burr, 2004; Zwerling et al., 1993). Exploring the possible differences in pinch force perception between males and females can offer novel insight regarding gender differences with respect to how MSD develop, contributing to the development of improved preventive strategies that decrease the high rate of hand injuries in both genders. The higher prevalence of MSD in females is typically associated with gender differences attributable to differences in strength, force perception, and anthropometry (Cote, 2012). Anthropometry and differences in strength have been detailed in the past, as in studies showing that relative to males, females have a lower muscle strength and aerobic capacity (Bao & Silverstein, 2005; Hallbeck et al., 1992; Herring-Marler et al., 2014), have a higher proportion of type I fibers (Simoneau & Bouchard, 1989), have a longer half-relaxation time (Hicks & McCartney, 1996), have a smaller twitch force in whole muscles (Miller et al., 1993), and exert more effort and stronger muscle contractions for the same task (Haward & Griffin, 2002). Specifically, it has been shown that females have smaller hands (Kong et al., 2007; Petrofsky et al., 1980) and muscle cross-sectional areas than do males (Miller et al., 1993). These differences may explain the higher rate of hand MSD in females.

However, recent research regarding gender differences in force perception indicate that additional factors beyond strength and anthropometry differences may drive gender-dependent differences in rates of injury (Bao & Silverstein, 2005; Herring-Marler et al., 2014; Song et al., 2006). Specifically, many authors have studied the effects of gender on force reproduction errors, but the results have been inconsistent. A previous study by Bao demonstrated that gender had no statistically significant influence on the validity of a reproduced force when comparing an estimated force with a known palmer pinch force (Bao & Silverstein, 2005). However, Herring-Marler studied how a participant’s gender influenced his or her finger force estimation accuracy in a submaximal ramped force matching task and found a significant difference in target force matching, with males showing larger errors than females (Herring-Marler et al., 2014). In addition, another study showed significantly more force-matching errors in females than in males at 70° of knee joint extension (Song et al., 2006). Males have larger hand sizes (which may correspond to a larger amount of skin contact) and larger muscle cross-sectional areas (which may correspond to a larger number of proprioceptive sensors) than females. Hence, we hypothesized that males more accurately reproduce pinching forces than do females.

As mentioned earlier, the most accurate estimation in pinch grip may occurs for lower force levels compared with other joints, and the results of previous studies about the effects of gender on force reproduction errors have been inconsistent. Therefore, the objective of this study was to explore how force level and gender impacted pinch force reproduction accuracy errors in normal participants.

## Methods

### Participants

Twenty healthy participants (10 females and 10 males, age 18.4 ± 1.2 years, weight 57.8 ± 10.8 kg, height 167.1 ± 6.9 cm, all right-handed) participated in this study. Hand dominance for the participants was determined based upon which hand was used to write. The exclusion criteria were as follows: (a) a prior hand surgery and (b) the presence of hand pain or a hand pathology. Participants were informed of both study objectives and experimental procedures, and written consent forms were signed by them. Authorization to perform this research was granted by the ethics review board of our university.

### Apparatus

An electronic digital force dynamometer (pinch analyzer; Kjyl Technologies, Beijing, China) was utilized for all strength and force reproduction testing. Manufacturer calibration settings were used, and the instrument was validated before experimentation. The adjustable pinch span of this device was set at 2 cm for all testing, and sampling frequency was maintained at 100 Hz. Based on the pinch analyzer, a protocol was developed to measure pinch force perception.

### Protocol

**Figure 1.**
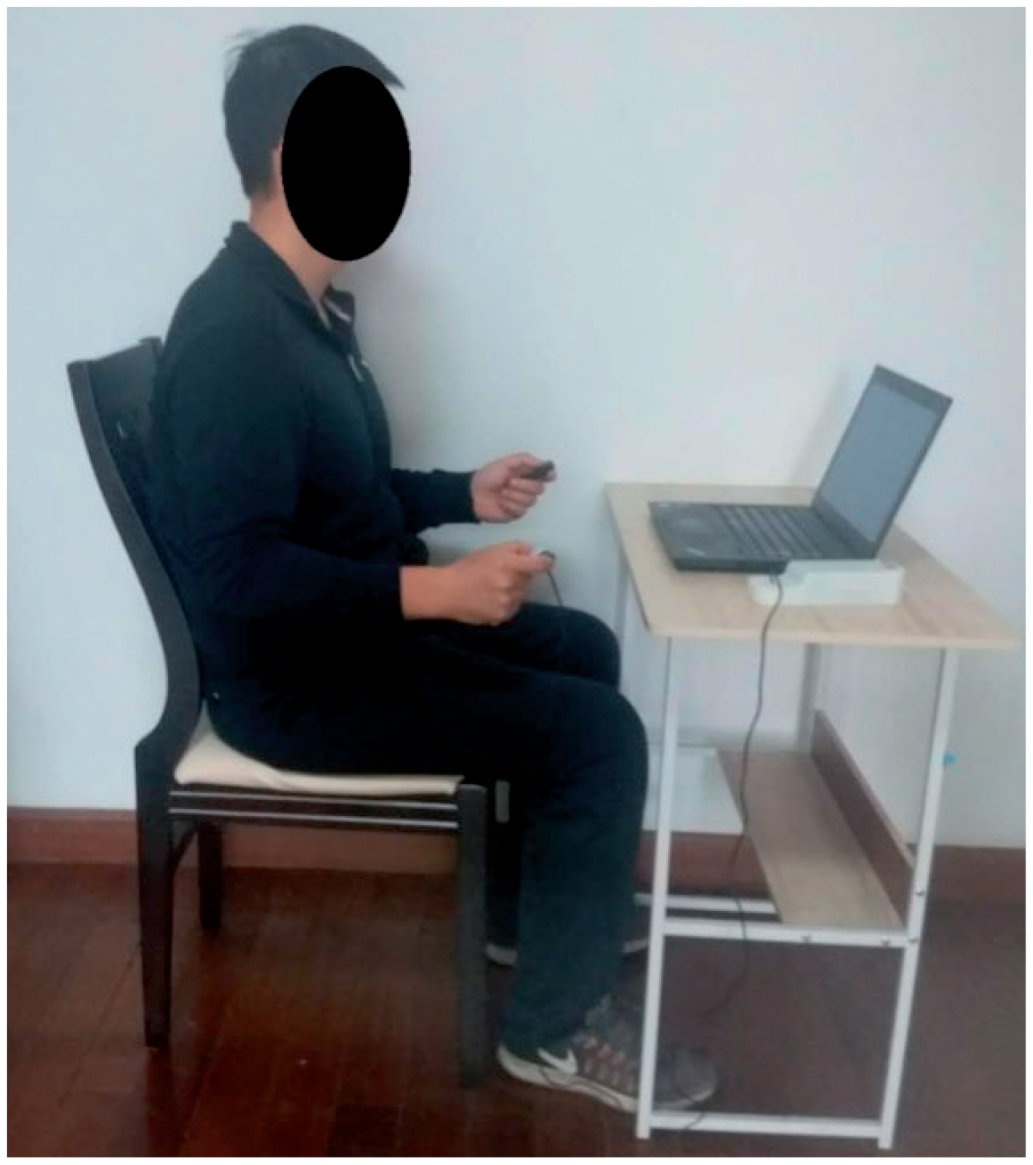
The Standardized Body and Monitor Positioning Used for the Pinch Force Reproduction Measurements.

Auditory distractions were minimized by conducting the study in a quiet room (Lubiatowski et al., 2013; Nozoe et al., 2011). The participants sat in a chair that was located approximately 60 cm in front of a 14-in. LCD monitor, after which a whole-body posture was adopted, in line with the American Society of Hand Therapists guidelines: The upper arm was positioned vertically, the elbow was flexed at 90°, and the forearm and wrist were placed in neutral positions (van den Noort et al., 2014). The participants performed isometric pinching tasks with the tip pinch. The tip pinch was chosen because this pinch configuration is often required for precision tasks (Dong et al., 2006). The tip pinch involves moving the tip of the thumb to the tip of the index finger with the other fingers fully flexed (Figure 1; Visser et al., 2003). Participants were instructed to maintain this configuration for the duration of testing and were able to see their pinch force output on the computer screen. Data were acquired and processed with a PC using a customized MVIC test program and force reproduction task program (Kjyl Technologies).

#### Maximal Voluntary Isometric Contraction

The participants performed warm-up activity prior to testing. They were requested to use a pinch grip and to exert a maximal pinch force on the dynamometer. This test was repeated twice, and the maximum value was recorded as the pinch strength (Bao & Silverstein, 2005). To reduce the impact of fatigue on study outcomes, participants were able to rest for 3 minutes between tests.

#### Force Reproduction Task

**Figure 2.**
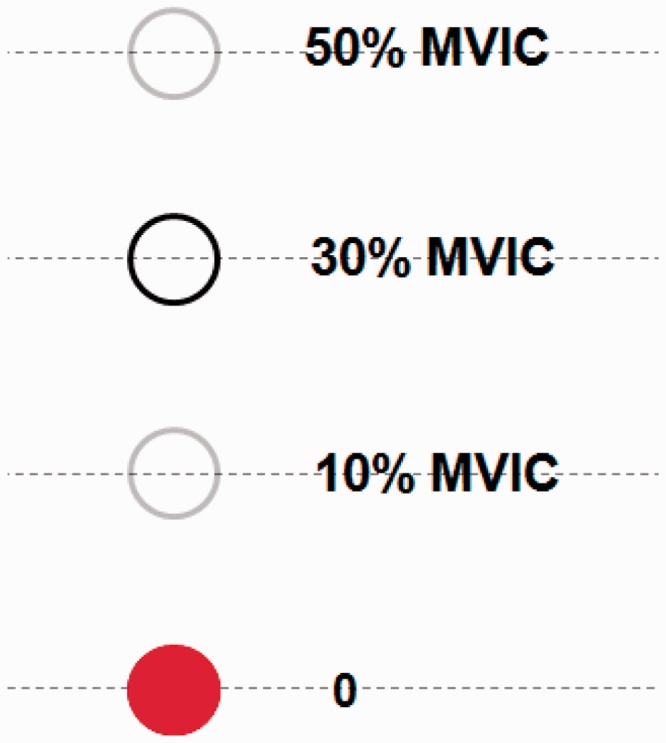
Schematic of the Computer Display, Which Was Used to Guide the Participant to the Target Force. MVIC = maximal voluntary isometric contraction.

The ability of participants to accurately reproduce a target force was used to measure force perception. Participants first watched a video describing the task. A custom C++ program was then used, wherein participants were shown a black dot symbolizing target force in a given trial. Instantaneous pinch force was represented on the same screen by a red dot (Figure 2). The participants were required to exert a target force, *T*, for 3 seconds with a pinch grip, and they were asked to memorize the level of force applied. Participants were then instructed to close their eyes and relax for 3 seconds, after which they were asked to replicate this same force without any visual feedback. Participants were instructed to use their other hand to press a trigger when they believed that they had successful replicate the prior force, after which the exerted force (*R*) was recorded by the computer. Participants were then told to relax again. This task was repeatedly practiced until the participant comprehended and was comfortable with the procedure. Twelve different forces ranging from 5% to 60% MVIC in increments of 5% MVIC were replicated by the participant, and three repeated contractions per force level were performed. Target force order was randomized. Participants were allowed to rest for 30 seconds after each trial to minimize fatigue, and they additionally rested 2 to 3 minutes following the completion of five trials to ensure continued attentiveness (Marini et al., 2017).

### Statistical Analyses

Three dependent variables were assessed with respect to errors in force sensing: absolute error (AE; Bang et al., 2015; Baray et al., 2014; Duzgun et al., 2011; Han & Lee, 2014; Maenhout et al., 2012; Trousset et al., 2018; Vila-Cha et al., 2011), constant error (CE; Bennell et al., 2005; Knox & Hodges, 2005; Lonn et al., 2000; Salgado et al., 2015; Winter et al., 2005), and variable error (VE; Ettinger et al., 2017; Mazuchi et al., 2018; Vafadar et al., 2015). While AE provides insight into overall error, CE offers insight into error direction (i.e., overestimation or underestimation), and the VE corresponds to how the error varied over the course of series of trials, thus reflecting the precision of a given participant. These three values were normalized to target force values in an effort to analyze error relative to MVIC regardless of target force magnitude, yielding normalized AE (NAE), normalized CE (NCE), and normalized VE (NVE), respectively (Trousset et al., 2018). These parameters were calculated as follows:

(1) $$\mathrm{AE}=\frac{\sum_{i=1}^{3}\left|R_{i}-T\right|}{3},\;\left(i=1\text{, }2,3\right)$$

(2) $$\mathrm{CE}=\frac{\sum_{i=1}^{3}\left(R_{i}-T\right)}{3},\;\left(i=1\text{,}2,3\right)$$

(3) $$\mathrm{VE}=\frac{\sum_{i=1}^{3}\sqrt{{\left(R_{i}-\bar{R}\right)}^{2}}}{3},\;\left(i=1,\;2,\;3\right)$$

(4) $$\mathrm{NAE}=\frac{\sum_{i=1}^{3}\left|R_{i}-T\right|}{3\cdot T}\times 100\%,\;\left(i=1\text{,}2,3\right)$$

(5) $$\mathrm{NCE}=\frac{\sum_{i=1}^{3}\left(R_{i}-T\right)}{3\cdot T}\times 100\%,\;\left(i=1\text{,}2,3\right)$$

(6) $$\mathrm{NVE}=\frac{\sum_{i=1}^{3}\sqrt{{\left(R_{i}-\bar{R}\right)}^{2}}}{3\cdot T}\times 100\%,\;\left(i=1,\;2,\;3\right)$$

where *R_i_* is the reproductive force for the *i*th trial, *T* represents target force, and R(_) represents mean across three trials.

An independent samples *t* test was used to determine the MVIC differences between genders. One-sample *t* tests were performed to compare the NCE to zero for each force level and to identify the trials in which subjects generated excessively high or low forces. To assess the effect of the force levels (12 different forces ranging from 5% to 60% MVIC in increments of 5% MVIC) and gender (males and females) on the NAE and NVE, mixed-model analyses of variance were performed. Gender was included as a between-subject factor and force level as a within-subjects factor. The significant interaction and main effects were reassessed with additional comparisons, and Bonferroni-corrected post hoc comparisons were used for multiple comparisons. Statistical analyses were performed with SPSS 22.0 (IBM, Armonk, NY, USA) package. Data are represented as means ± standard error, and *p* < .05 was the significance threshold.

## Results

### MVIC, AE, CE, and VE

**Figure 3.**
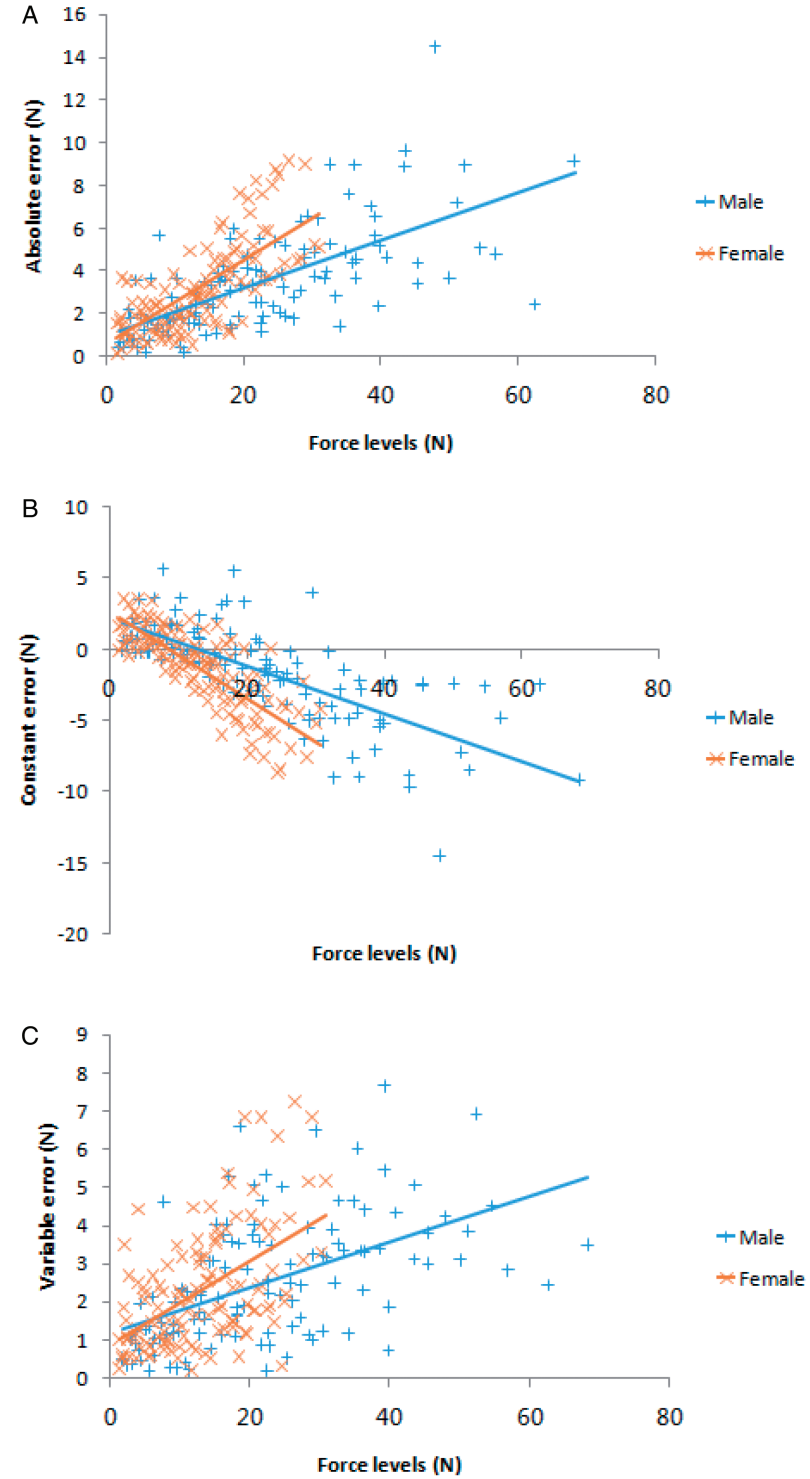
(A) AE, (B) CE, and (C) VE of the individual data, corresponding to an individual’s accuracy, direction, and precision as a function of their gender and generated force levels.

The results showed significantly higher pinch forces in the males (68.5 ± 23.6 N) than in the females (40.2 ± 8.3 N), *t*(18) = 3.59, *p* = .004. The MVIC of the females was 70.4% less than that of the males. The individual data of the AE, CE, and VE are presented in Figure 3.

### NAE and NVE

**Figure 4.**
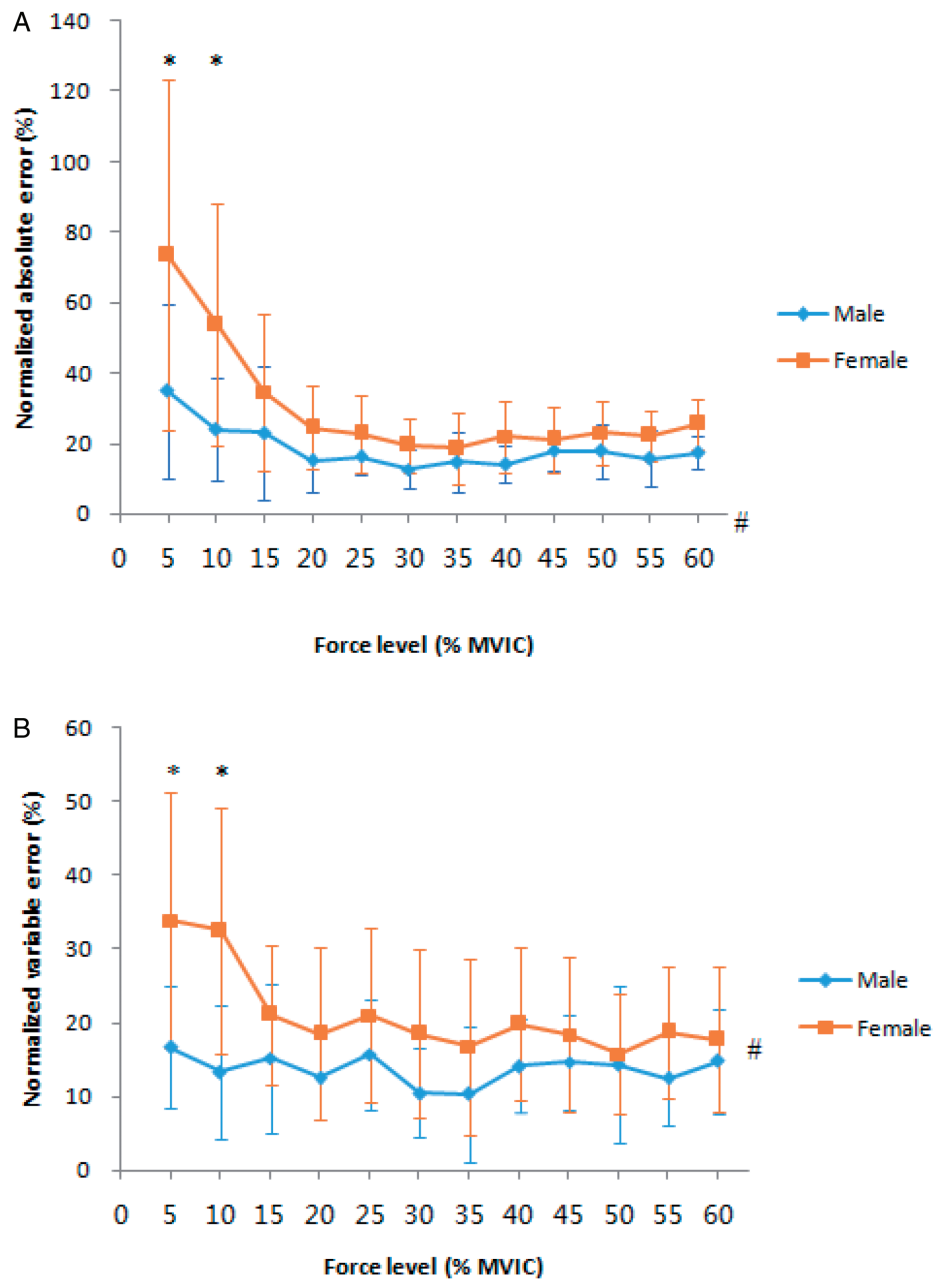
A: NAE, an assessment of overall force reproduction error, as a function of gender and force levels. B: NVE, which represents error variability across trials and more generally, the precision of the individuals’ performance. Error bars: standard errors of the mean across participants (*p < .05, significant within-subject differences. ^#^*p* < .05, significant between-subject differences). MVIC = maximal voluntary isometric contraction.

There was a significant main effect for gender, NAE: *F*(1, 18) = 9.18, *p* = .007 or NVE: *F*(1, 18) = 4.69, *p* = .044. The results showed that the males produced a significantly lower NAE and NVE than did the females. There was a significant main effect for the force levels, NAE: *F*(1, 18) = 11.63, *p* = .003 or NVE: *F*(1, 18) = 5.01, *p* = .038. The results showed a significantly higher NAE and NVE at 5% and 10% MVIC than at other force levels (*p* < .05). Mixed-model analysis of variance revealed that the interaction between gender and force levels was not significant for the NAE, *F*(1, 18) = 2.77, *p* = .114, or NVE, *F*(1, 18) = 3.02, *p* = .099 (Figure 4).

### Normalized CE

**Figure 5.**
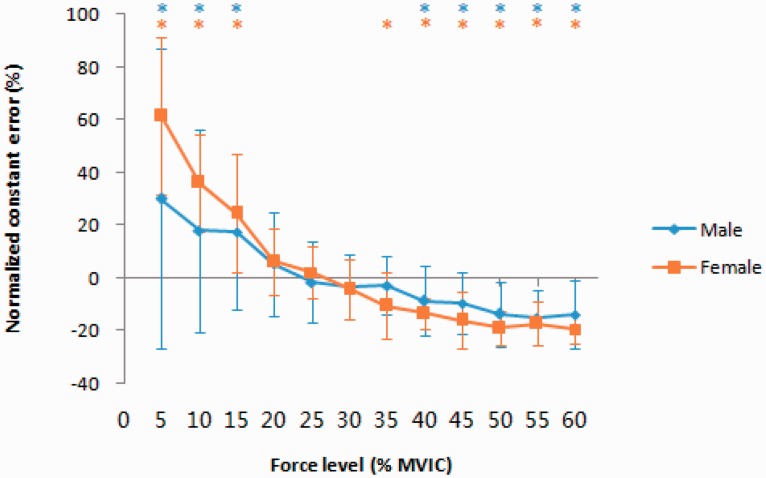
NCE, the Directionality of the Error of the Reproduced Force, as a Function of Gender and Force Levels. Error bars: standard errors of the mean across participants (males: *(cyan asterisk) *p* < .05, females: *(orange asterisk) *p* < .05). MVIC = maximal voluntary isometric contraction.

The results showed a significantly higher NCE for force levels as high as 15% MVIC, all *t*(9) > 2.45, *p* < .05, and a lower NCE for force levels equal to or higher than 35% MVIC (females) or 40% MVIC (males), all *t*(9) < −2.80, *p* < .05; the most accurate estimations occurred at approximately 20% to 35% MVIC, males: 20% to 40% MVIC, *t*(9) = −0.67 to −1.38 and females: 20% to 35% MVIC, *t*(9) = −1.04 to −1.02, all *p* > .05 (Figure 5).

## Discussion

A common way to investigate human force sensation is by performing force reproduction tasks, in which participants have to reproduce a previously experienced force (Maenhout et al., 2012; Mugge et al., 2015; Phillips & Karduna, 2017; Trousset et al., 2018). Regarding the performance of participants in force reproduction tasks, visual information, learning, memory, and proprioceptive feedback are the four major components that guide participants to the target force. In our study, the participants were asked to close their eyes to block any visual feedback. The target forces were repeated in a randomized order, which limited the effects of learning. To minimize the potential for any memory effects due to concentration difficulties, the study was performed in a quiet room and with short and consistent intervals (3 seconds) between the production and reproduction of the target force, which were controlled. Thus, we believe that proprioceptive feedback related to force sensation contributed to the force reproduction accuracy.

Our findings show that smaller force values (i.e., 5%–15% MVIC) were reliably overproduced, whereas higher forces (i.e., 40%–60% MVIC) were reliably underproduced. Estimates were most accurate at 20% to 35% MVIC (females) and 20% to 40% MVIC (males). This finding is concordant with that reported in one prior study (Kumar & Simmonds, 1994) in which the power grip, key pinch, and stoop lifting activity were utilized to assess perception reliability and accuracy. Perceived forces at 60% and 80% MVIC were lower, and the perceived forces at 20% MVIC were higher than the actual MVIC values. At 40% effort, perceived effort and objective effort were not statistically different for all three activities (Kumar & Simmonds, 1994). Similar findings were observed for the index finger (Walsh et al., 2011), elbows (Jones & Hunter, 1982), knees (West et al., 2005), and chest press exercises (Jackson & Dishman, 2000); the most accurate estimations occurred at approximately 50% MVIC. The overestimation of lower forces and underestimation of higher forces have been well described and discussed earlier (Jackson & Dishman, 2000; Jones & Hunter, 1982; Kumar & Simmonds, 1994; Shergill et al., 2003; Walsh et al., 2011; West et al., 2005). Therefore, we try to focus on the force levels corresponding to the most accurate estimates with the pinch grip.

In this study, the most accurate isometric force estimates were at approximately 20% to 35% MVIC. This is slightly different from prior findings of 40% to 50% MVIC (Jackson & Dishman, 2000; Jones & Hunter, 1982; Kumar & Simmonds, 1994; Walsh et al., 2011; West et al., 2005). Our results were primarily consistent with a hypothesis, wherein the most accurate estimation in pinch grip occurs for lower force levels (approximately 20%–35% MVIC) compared with other joints (40%–50% MVIC). The disparities between our results and prior findings may be due to the use of different joints. Each joint has physiological and anatomical differences as well as differences in numbers of mechanoreceptors. Relative to other joints, the fingers are controlled with greater precision by the tendons attached to the muscles. Because finger movements require the coordination of many tendons, which are generally not as oxygenated as muscles, they easily lead to fatigue. While we observed the participants exert and control forces, we found it to be challenging to accurately maintain a target force of more than 60% MVIC. This may also be related to the coordination of forces between the thumb and the index finger in the pinch grip. Prior studies on the targeting errors and variability with regard to submaximal forces of the fingers proposed that a lack of coordination of the digits results in increased errors (Sharp & Newell, 2000). As force increases, the difficulty of pinch grip coordination increases. This increase results in increased errors and variability (Rubley et al., 2003). The greater errors in higher force levels might also be related to the increased motor noise associated with larger motor commands (Harris & Wolpert, 1998). The larger the motor command, the greater the variability of the motor output, which may hinder the estimation of the sensory feedback, resulting in greater perceptual variability.

For ergonomic reasons, tools are typically designed according to the optimal force level, maximizing the accuracy of predictions and production of pinch forces. Our results, in which the most accurate estimation occurred at approximately 20% to 35% MVIC, might be used to scale the forces that operators of hand tools need to exert by, for example, adjusting the handle length so that operators perceive forces more accurately. In addition, our results minimize the risk of pinch muscle fatigue and hand MSDs.

Unlike the results in previous studies (Bao & Silverstein, 2005; Herring-Marler et al., 2014), our results are consistent with the hypothesis that males more accurately (AE) and consistently (VE) reproduce pinching forces than do females. The discrepancy across previous studies in the accuracy of the force performance between genders may be attributed to many factors, such as the experimental design, target force, dependent variables, age of the participants, and joint used (Bao & Silverstein, 2005; Herring-Marler et al., 2014; Song et al., 2006). More studies need to be conducted to understand how gender is correlated with force reproduction errors. Our results can be explained by the findings that information on the muscle forces and interaction forces are identified by proprioceptive sensors (the muscle spindles and the Golgi tendon organs) in the muscles and tactile sensors in the skin (Onneweer et al., 2013). Previous articles have indicated that proprioceptive sensors are sensitive to high force levels, while tactile sensors are sensitive to low force levels (Onneweer et al., 2013). Males have more muscle mass than females, and the number of proprioceptive sensors is larger in muscles with larger cross-sectional areas (Banks, 2006; Kokkorogiannis, 2004). Therefore, there may be more proprioceptive sensors in males than in females. Tests for tactile sensitivity did not demonstrate any differences in cutaneous sensitivity between the pads of the thumb and index finger (Weinstein, 1968). Thus, activation of the additional tactile sensors in the finger pad might be dependent on an increase in skin contact. Males have a larger hand (Kong et al., 2007; Petrofsky et al., 1980) and a larger area of skin contact than do females while exerting a force. In addition, males exhibit improved balance and control than females while exerting forces. Thus, males more accurately and consistently reproduce pinching forces than do females. Although employing constant movements with lower accuracy and consistency in performance may only induce minimal stress on the fingers, if these forces are applied for extended time periods, a significant injury may develop. This, together with prior findings, offers new insight into the causes of higher MSD rates in females than in males and can assist in the formulation of preventive strategies aimed at reducing risk of injury. A scatter plot (Figure 3) showing the presented force (N) on the horizontal axis and the error on the vertical axis documented that the effect of the gender is not a consequence of the different force range presented to males and females.

## Conclusion

Our study showed that males more accurately and consistently reproduce pinching forces than do females, and the most accurate estimation occurred at approximately 20% to 35% MVIC. This finding can help us better understand the reasons for the higher rate of MSD in females than in males and to develop tools and preventive strategies that decrease the high rate of hand injuries in both genders.
